# Wnt4 is a novel biomarker for the early detection of kidney tubular injury after ischemia/reperfusion injury

**DOI:** 10.1038/srep32610

**Published:** 2016-09-07

**Authors:** Shi-Lei Zhao, Shi-Yao Wei, Yu-Xiao Wang, Tian-Tian Diao, Jian-Si Li, Yi-Xin He, Jing Yu, Xi-Yue Jiang, Yang Cao, Xin-Yue Mao, Qiu-Ju Wei, Yu Wang, Bing Li

**Affiliations:** 1 Department of Nephrology, 2nd Affiliated Hospital, Harbin Medical University, Harbin, People’s Republic of China; 2 Department of Nephrology, 1st Affiliated Hospital, Harbin Medical University, Harbin, People’s Republic of China

## Abstract

Earlier intervention after acute kidney injury would promote better outcomes. Our previous study found that Wnt proteins are promptly upregulated after ischemic kidney injury. Thus, we assessed whether Wnt4 could be an early and sensitive biomarker of tubular injury. We subjected mice to bilateral ischemia/reperfusion injury (IRI). Kidney and urinary Wnt4 expression showed an early increase at 3 hours and increased further at 24 hours post-IRI and was closely correlated with histopathological alterations. Serum creatinine slightly increased at 6 hours, indicating that it was less sensitive than Wnt4 expression. These data were further confirmed by clinical study. Both kidney and urinary Wnt4 expression were significantly increased in patients diagnosed with biopsy-proven minimal change disease (MCD) with tubular injury, all of whom nevertheless had normal estimated glomerular filtration rate (eGFR) and serum creatinine. The increased Wnt4 expression also strongly correlated with histopathological alterations in these MCD patients. In conclusion, this is the first demonstration that increases in both kidney and urinary Wnt4 expression can be detected more sensitively and earlier than serum creatinine after kidney injury. In particular, urinary Wnt4 could be a potential noninvasive biomarker for the early detection of tubular injury.

Acute kidney injury (AKI) is an important clinical problem as a complication with a high incidence of morbidity and mortality^1^. Despite many improvements in our understanding of AKI, there are few therapies for patients with AKI. Our previous study has demonstrated that an earlier administration of hematopoietic stem cells after kidney injury promotes a better outcome^2^. It is imperative to establish an earlier diagnosis to enable earlier intervention. Currently, the diagnosis of AKI depends on serum creatinine, which increases significantly only after substantial kidney injury occurs and then after a significant time delay^3^. In the early phase of AKI, changes first occur at the cellular and molecular levels before serum creatinine is elevated. These changes ultimately lead to renal dysfunction and structural injury^4^. Using serum creatinine as a marker of AKI could delay effective treatment at the early stages of AKI; thus, identifying reliable earlier biomarkers is extremely urgent^5^. There has been substantial interest in the discovery of biomarkers for identifying AKI at its earliest stage, when interventions might be more effective. Early diagnosis plays a key role in the successful treatment of AKI^6^. Therefore, identifying a sensitive biomarker for early detection of AKI is highly desirable.

Wnt/β-catenin signaling is an evolutionarily conserved cellular signaling system that plays a key role in diverse aspects of biological processes, such as tissue homeostasis, organogenesis, and the pathogenesis of many human diseases^7^. The variable expression of Wnt/β-catenin has been implicated in many types of kidney disease^8^,^9^,^10^. Our previous study found that Wnt proteins are promptly and dramatically upregulated after ischemic kidney injury^11^. Among the Wnt family, Wnt4 is expressed in epithelial progenitors in developing kidney and is associated with tubulogenesis^12^. Wnt4 knockout mice show a complete lack of tubular development, despite the aggregation of cells at ureteric bud tips and initially normal ureteric bud branching, which indicates that Wnt4 is necessary for tubular induction^13^. Wnt4 expression is upregulated in the injured renal tubules at a very early stage after ischemia/reperfusion injury (IRI)^13^,^14^. Epithelial Wnt4 is also observed in murine folic acid nephropathy, which is another experimental model of AKI^15^. These experiments demonstrated that increased Wnt4 expression at early stage might be an early indicator of kidney injury, except it may play a critical role during the repair and regeneration process in AKI. Thus, we investigated Wnt4 expression in a mouse model of IRI and in patients with a normal estimated glomerular filtration rate (eGFR) who were diagnosed with biopsy-proven minimal change disease (MCD) with or without tubular injury.

In the present study, in both IRI mice and MCD patients with tubular injury, we show that increased levels of Wnt4 can be detected in the urine and serve as a potential noninvasive biomarker for the early detection of tubular injury.

## Results

### Kidney function and histological changes in IRI mice

**Figure 1:**
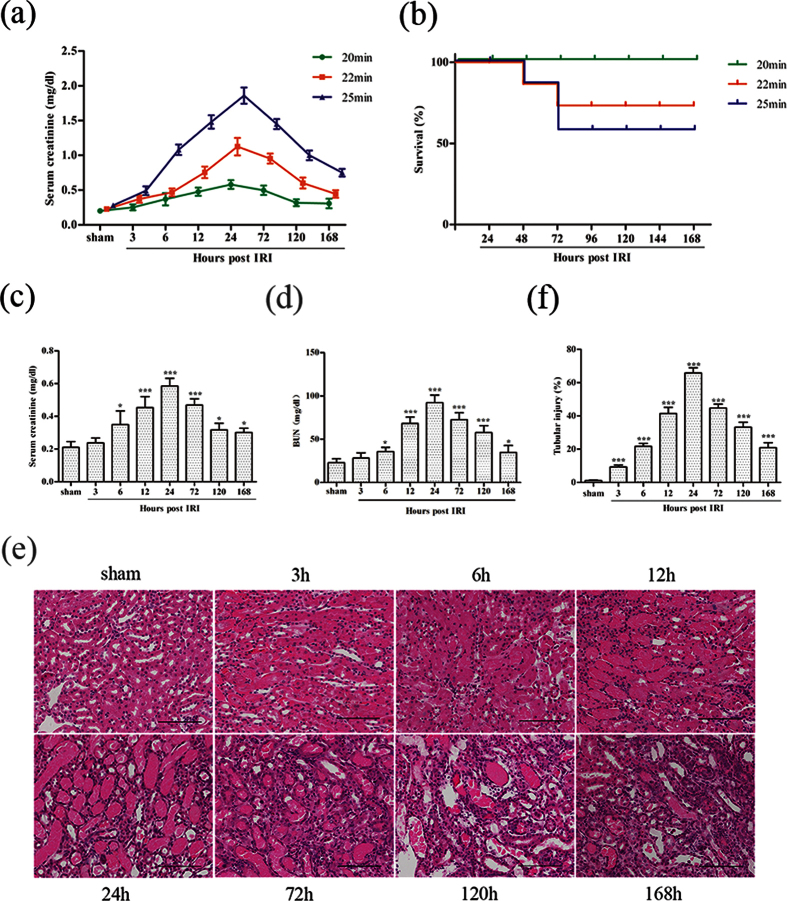
Characterization of mice after IRI. **(a)** Serum creatinine at different time points in sham-operated and IRI mice. (**b)** Survival (%) of IRI mice. (**c)** Serum creatinine and (**d**) BUN level at different time points after 20 minutes of bilateral renal ischemia or sham operation. (**e)** Representative images of HE-stained sections at different time points after 20 minutes of bilateral renal ischemia (magnification, 200x). Scale bar, 100 μm. (**f)** Renal tubular injury scores from HE staining. The data are expressed as the mean ± SD. *P < 0.05, ***P < 0.001 versus the sham group (n = 8 in each group).

To find a better early kidney injury model, we subjected mice to bilateral IRI with different ischemic times (20, 22 and 25 minutes). Bilateral kidney IRI induced a mild increase in serum creatinine at 6 hours and peaked at 24 hours (Fig. 1a). The longest period of ischemia (25 minutes) induced the most serious kidney injury, and approximately 50% of the mice died at 72 hours after reperfusion (Fig. 1b). Thus, we chose the mildest injury group (20 minutes) to investigate the early indicators of AKI. The extent of kidney damage was assessed by histological alteration and changes in serum creatinine and blood urea nitrogen (BUN) at different time points simultaneously (Fig. 1c–e). As early as 3 hours after reperfusion, kidney tissue showed focal tubular injury with brush border loss and cellular swelling. The most serious injury was observed at 24 hours after IRI (Fig. 1e). Kidney tubular injury was scored by semiquantitative histopathological analysis (Fig. 1f). In contrast, serum creatinine and BUN level slightly increased at 6 hours and peaked at 24 hours after reperfusion in mice subjected to 20 minutes of bilateral renal ischemia but did not significantly change at 3 hours (Fig. 1c,d). These data further demonstrate that serum creatinine has low sensitivity in detecting kidney damage in the early phase of kidney tubular injury. Therefore, identifying a sensitive biomarker for the early detection of kidney tubular injury is highly desirable.

### Wnt4 is markedly upregulated in kidney after IRI, which correlates with kidney tubular injury and KIM-1 expression

**Figure 2:**
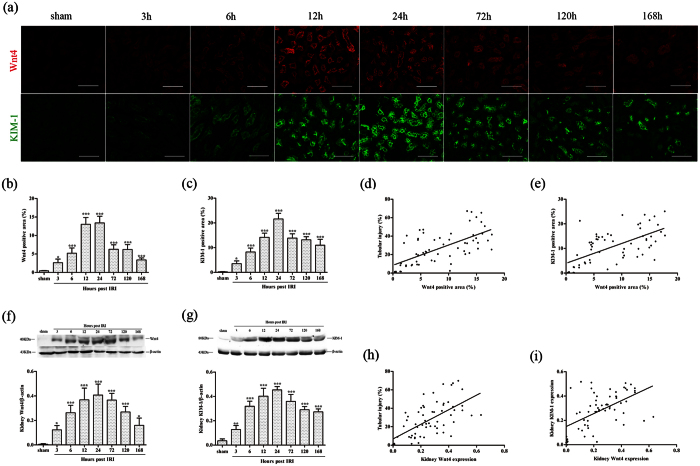
Kidney Wnt4 expression markedly increases in IRI mice, in correlation with tubular injury and kidney KIM-1 expression. (**a)** Representative immunofluorescence images of kidney Wnt4 and KIM-1 in the IRI mice at different time points and in sham-operated mice (magnification, 200x). Scale bar, 100 μm. (**b)** Quantification of the immunofluorescence staining of Wnt4 in each group. (**c)** Quantification of the immunofluorescence staining of KIM-1 in each group. (**d)** Correlation between kidney Wnt4 expression and tubular injury. (**e)** Correlation between kidney Wnt4 and KIM-1 by immunofluorescence staining. (**f)** Western blot analysis and quantification of kidney Wnt4 expression in each group. (**g)** Western blot analysis and quantification of kidney KIM-1 expression in each group. (**h)** Correlation between kidney Wnt4 expression and tubular injury. (**i)** Correlation between kidney Wnt4 and KIM-1 expression by western blot. The gels were run under the same experimental conditions. *P < 0.05, **P < 0.01, ***P < 0.001 versus the sham group (n = 8 in each group).

The Wnt pathway is activated during repair and regeneration in animal models of acute ischemic injury^11^. According to our previous study and other publications, the Wnt4 gene is activated in the early stage of kidney injury and dermal wound^11^,^16^. In the present study, we first investigated the changes in Wnt4 expression in the kidney throughout the process after IRI. As shown in Fig. 2a, enhanced Wnt4 expression occurred at injured tubules as early as 3 hours after IRI by immunofluorescence staining, and reached a peak at 12 and 24 hours that persisted for at least 7 days after IRI. The same result is expressed graphically in Fig. 2b. Similar to Wnt4, KIM-1 expression slightly increased at 3 hours and peaked at 24 hours (Fig. 2a,c). Renal Wnt4 expression, as shown by immunofluorescence was positively correlated with kidney tubular injury (r = 0.66, P < 0.001; Fig. 2d). In addition, renal Wnt4 expression was also highly correlated with KIM-1 expression (r = 0.65, P < 0.001; Fig. 2e). We confirmed this finding by western blot. As shown in Fig. 2f, markedly increased Wnt4 expression was observed at 3 hours, which was further increased at 12 and 24 hours after IRI, consistent with immunofluorescence staining. KIM-1 expression showed a similar trend to Wnt4 in western blots (Fig. 2g). Western blot again confirmed that kidney Wnt4 expression was positively correlated with kidney tubular injury (r = 0.64, P < 0.001; Fig. 2h) and kidney KIM-1 expression (r = 0.56, P < 0.001; Fig. 2i). These data indicated that Wnt4 might be a more sensitive biomarker for early detection of tubular injury than serum creatinine in IRI mice.

### Enhanced expressed Wnt4 was localized in both the injured proximal and distal tubules following IRI, and almost all TUNEL positive cells and majority of Ki67 positive cells co-labeled with Wnt4

**Figure 3:**
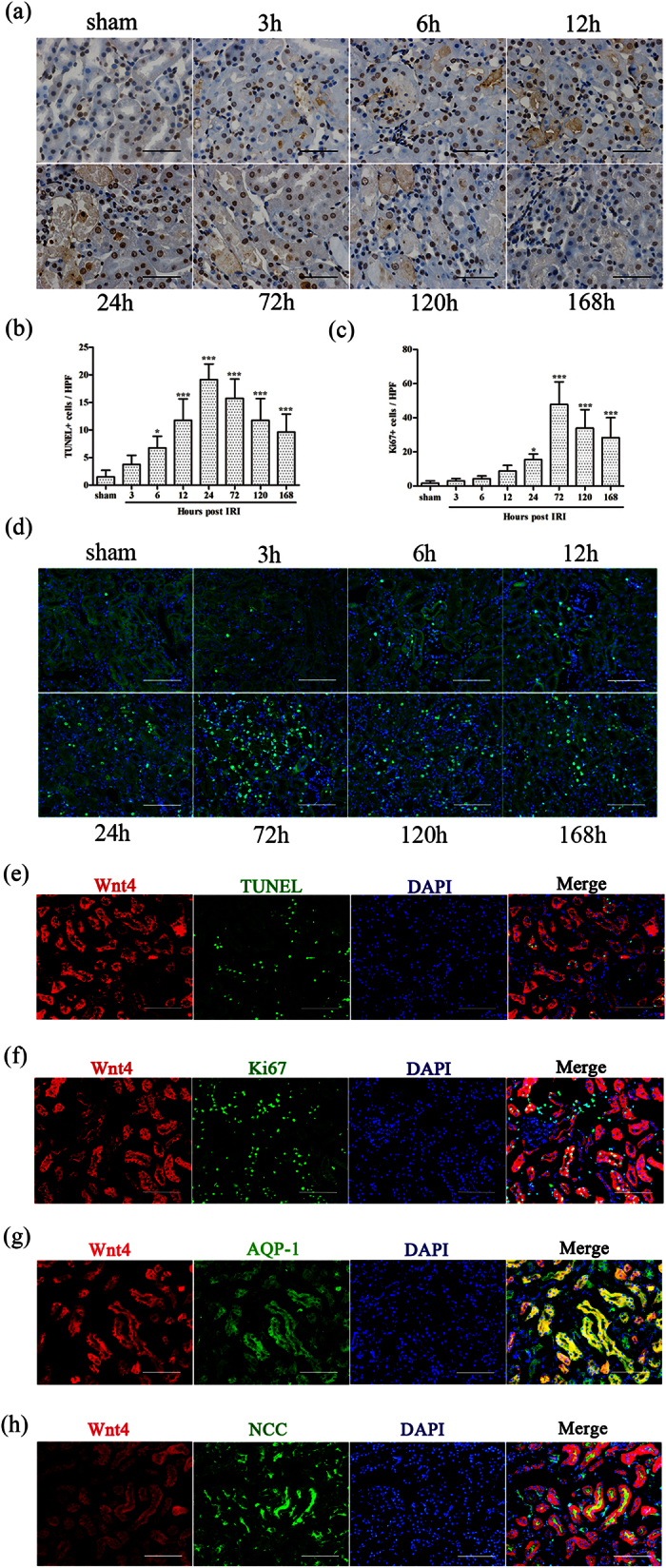
Enhanced expressed Wnt4 was localized in both the injured proximal and distal tubules following IRI, and almost all TUNEL positive cells and majority of Ki67 positive cells co-labeled with Wnt4. **(a)** Representative images of TUNEL staining (brown) in each group (magnification, 400x). (**b)** Quantification of TUNEL-positive cells in each group. (**c)** Quantification of Ki67-positive cells in each group. (**d)** Representative Ki67 immunofluorescence images in each group (magnification, 200x). (**e)** Co-staining for TUNEL and Wnt4 (magnification, 200x). **(f)** Co-staining for Ki67 and Wnt4 (magnification, 200x). (**g)** Co-staining for Wnt4 and segment-specific tubular markers, AQP1 (**h**) and NCC, in injured kidneys 12 hours after IRI (magnification, 200x). All scale bars, 100 μm. *P < 0.05, **P < 0.01, ***P < 0.001 versus the sham group (n = 8 in each group).

We also investigated the role of Wnt4 in reflecting either the injury phase or regenerative phase of renal tubules after IRI using terminal deoxynucleotidyl transferase dUTP nick-end labeling (TUNEL) and the pan-cell-cycle marker Ki67. As shown in Fig. 3a,b, the number of TUNEL positive apoptotic cells increased rapidly and peaked at 24 hours after IRI, followed by a gradual decrease over time. In addition, Ki67 positive cells gradually increased in injured tubules, peaking at 72 hours after IRI (Fig. 3c,d). Furthermore, almost all TUNEL positive cells and majority of Ki67 positive cells costained with Wnt4 (Fig. 3e,f), indicating that Wnt4 may undergo rapid activation after kidney injury and then involved in the repair and regeneration, as reported by our previous work and other publication^11^,^13^. To investigate the location of Wnt4 expression in different tubular segments, we performed co-staining with Wnt4 and the segment-specific tubular markers aquaporin-1 (AQP1) for proximal tubules and thiazide-sensitive NaCl cotransporter (NCC) for distal tubules. As shown in Fig. 3g,h, Wnt-4 was co-localized with both AQP1 and NCC. These results suggested that Wnt4 was activated in the injured tubules (both proximal and distal tubules). These results are in contrast to the behavior of KIM-1, which was simply secreted by injured proximal tubules^17^.

### Urinary Wnt4 is detected during the early stage after IRI and correlates with tubular injury and kidney Wnt4 expression

**Figure 4:**
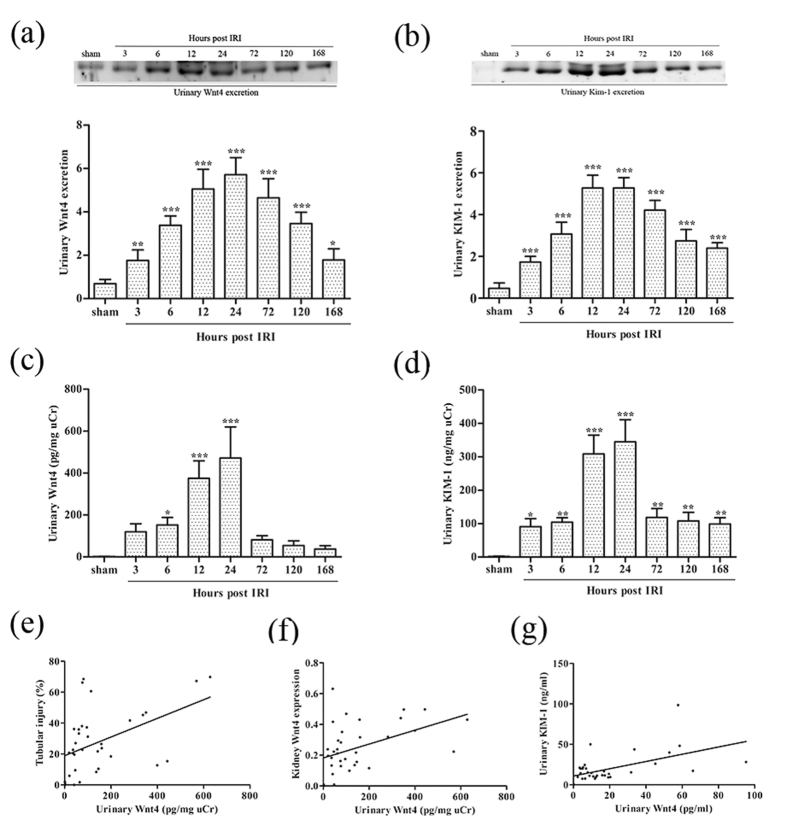
Urinary Wnt4 excretion is significantly elevated early in IRI mice, in correlation with tubular injury and urinary KIM-1. **(a)** Western blot analysis and quantification of urinary Wnt4 in each group. (**b)** Western blot analysis and quantification of urinary KIM-1 in each group. (**c)** ELISA analysis of urinary Wnt4 normalized to uCr in each group. (**d)** ELISA analysis of urinary KIM-1 normalized to uCr in each group. (**e)** Correlation between urinary Wnt4 levels and tubular injury. (**f)** Correlation between urinary Wnt4 excretion and kidney Wnt4 expression. (**g)** Correlation between urinary Wnt4 and KIM-1 excretion in mice. Cropped blots are shown (full-sized blots are presented in Supplementary Fig. S5). *P < 0.05, **P < 0.01, ***P < 0.001 versus the sham group (n = 8).

Because upregulated Wnt4 expression in the kidney was confirmed by western blot and immunofluorescence, we further investigated whether urinary Wnt4 could be detected at an early stage after IRI. As shown in Fig. 4a, urinary Wnt4 was significantly elevated at 3 hours compared to sham mice. It continued to increase at 6, 12 and 24 hours after IRI. These results were confirmed by ELISA (Fig. 4c). Simultaneously, we evaluated urinary KIM-1 by western blot and ELISA. It increased at 3 hours and peaked at 12 and 24 hours (Fig. 4b,d), similar to urinary Wnt4. In addition, the urinary Wnt4 level was strongly associated with the severity of kidney tubular injury (r = 0.49, P = 0.0028; Fig. 4e). The excretion of urinary Wnt4 was also closely associated with kidney Wnt4 expression (r = 0.45, P = 0.0059; Fig. 4f). Furthermore, urinary Wnt4 level was positively correlated with urinary KIM-1 level in IRI mice, as assessed by ELISA (r = 0.46, P = 0.0055; Fig. 4g). These data indicate that urinary Wnt4 might be a non-invasive biomarker for the early detection of kidney tubular injury after IRI.

### Kidney Wnt4 is upregulated in MCD patients with tubular injury and normal eGFR

**Figure 5:**
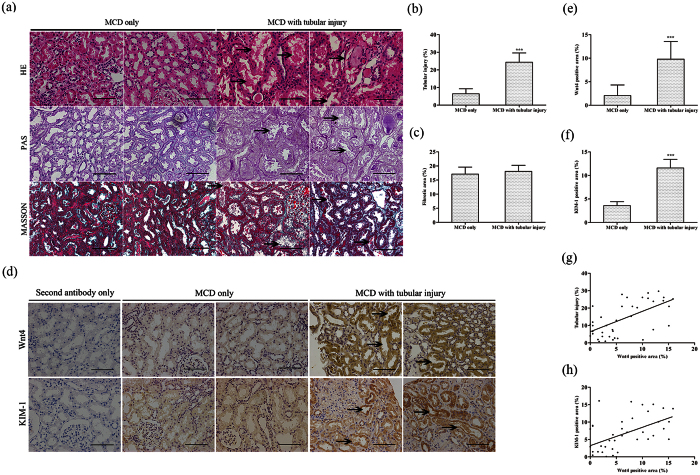
Kidney Wnt4 is upregulated in MCD patients with early AKI, similar to the expression of KIM-1. **(a)** Representative light microscopy images of HE, PAS and Masson-stained sections of the renal tissue in MCD patients with or without tubular injury (magnification, 200x). Scale bar, 100 μm. (**b)** Renal tubular injury score from HE staining. (**c)** Quantification of the fibrotic area from Masson staining. (**d)** Immunohistochemistry of kidney Wnt4 and KIM-1 (magnification, 200x). Scale bar, 100 μm. (**e)** Quantification of immunohistochemistry for Wnt4. (**f)** Quantification of immunohistochemistry for KIM-1. (**g)** Correlation between kidney Wnt4 expression and tubular injury in MCD patients. (**h)** Correlation between kidney Wnt4 and KIM-1 expression by immunohistochemistry. ***P < 0.001 versus MCD only.

We extended our observations from animal models to patients to evaluate whether kidney Wnt4 expression was elevated in early AKI patients. For this purpose, enrolled MCD patients diagnosed by clinic manifestation and renal pathology were separated with two groups: MCD without tubular injury (MCD only) and MCD with tubular injury by renal biopsy. The clinical characteristics of all patients are summarized in Table 1. The serum creatinine, blood urea nitrogen (BUN), eGFR and other physical and biochemical indicators were similar between the groups (Table 1). The representative images of hematoxylin-eosin (HE), periodic acid-Schiff (PAS) and Masson staining from MCD patients are presented in Fig. 4a. In the biopsy specimens from the MCD patients with tubular injury, we observed marked injury to the renal tubules (Fig. 5a,b). Masson staining revealed no differences in the fibrotic areas between the two groups (Fig. 5c). Increased Wnt4 expression was obviously observed in the renal tubular epithelia of MCD patients with tubular injury (Fig. 5d,e). A similar tendency occurred for KIM-1 expression (Fig. 5d,f). Furthermore, kidney Wnt4 expression in MCD patients was positively correlated with tubular injury (r = 0.59, P < 0.001; Fig. 5g). There was also a strong correlation between kidney Wnt4 and KIM-1 expression (r = 0.53, P < 0.001; Fig. 5h). These data suggest that enhanced kidney Wnt4 expression could be an earlier and more sensitive indicator of tubular injury than eGFR and serum creatinine.

### Urinary Wnt4 is elevated in MCD patients with tubular injury but normal SCr

**Figure 6:**
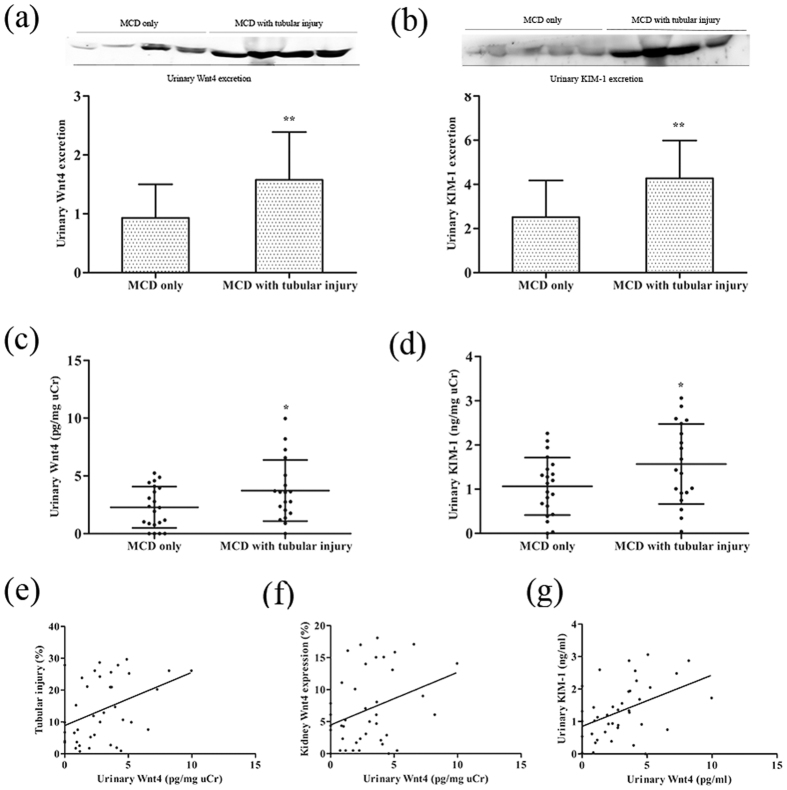
Urinary Wnt4 is elevated in MCD patients with early AKI but normal serum creatinine, similar to KIM-1. **(a)** Western blot analysis and quantification of urinary Wnt4 excretion in MCD patients with or without tubular injury. (**b)** Western blot analysis and quantification of urinary KIM-1 excretion in MCD patients with or without tubular injury. (**c)** ELISA analysis of urinary Wnt4 normalized to uCr in each group. (**d)** ELISA analysis of urinary KIM-1 normalized to uCr in each group. (**e)** Correlation between urinary Wnt4 level and tubular injury. (**f)** Correlation between urinary Wnt4 excretion and kidney Wnt4 expression (by immunochemical staining). (**g)** Correlation between urinary Wnt4 and KIM-1 excretion. Cropped blots are shown (full-sized blots are presented in Supplementary Fig. S5). *P < 0.05, **P < 0.01, versus MCD only.

Next, we investigated whether urinary Wnt4 could be detected in MCD patients with tubular injury. We measured urinary Wnt4 between the two groups by western blot and ELISA. Consistent with the changes in kidney Wnt4 expression, urinary Wnt4 was significantly elevated in the MCD patients with tubular injury compared with MCD-only patients (Fig. 6a); this tendency was confirmed by ELISA (Fig. 6c), and similar results were obtained for urinary KIM-1 excretion (Fig. 6b,d). The urinary Wnt4 level was positively correlated with kidney tubular injury (r = 0.40, P = 0.0115; Fig. 6e) and the degree of kidney Wnt4 expression by immunohistochemical staining (r = 0.33, P = 0.0372; Fig. 6f). These results were consistent with animal experiments. Furthermore, urinary Wnt4 excretion closely correlated with urinary KIM-1 level (r = 0.44, P = 0.0046; Fig. 6g). Notably, the urinary Wnt4 level was as sensitive as the urinary KIM-1 level for detecting early tubular injury in MCD patients. Altogether, our results show that the urinary Wnt4 level might be a non-invasive, earlier, and more sensitive biomarker for detection of kidney tubular injury than eGFR and serum creatinine.

## Discussion

AKI is a common and serious disease. Patients who develop AKI have markedly increased in-hospital mortality. However, AKI is largely asymptomatic in the initial stages. Although the diagnosis of AKI has improved in recent years, the criteria of acute kidney injury network and risk injury failure lost end-stage renal disease are both based on elevation of serum creatinine^18^, which is an insensitive, delayed and unreliable marker for AKI. Our study also indicated obvious renal tubular injury in IRI mice when serum creatinine was not increased dramatically. These limitations have instigated the search for novel proteins that are involved in the process of kidney tubular injury. In addition, the use of a single biomarker might not be adequate to determine whether AKI is due to various dysfunctional conditions or renal heterogeneity^19^. Therefore, sensitive and specific biomarkers for the early detection of AKI are urgently needed. The ideal biomarker for AKI should be fast and easy to measure, precise and accurate, and specific and sensitive for kidney injury^20^. In the present study, we found that renal Wnt4, as well as urinary Wnt4 expression, increased considerably at 3 hours and markedly increased at 24 hours post-IRI, in close correlation with histopathological alterations and KIM-1 expression. These data were further confirmed by a clinical study. Both kidney and urinary Wnt4 expression were markedly increased in MCD patients with tubular injury, accompanied by normal eGFR and serum creatinine. This is the first demonstration that both increased kidney and urinary Wnt4 expression can be detected earlier after IRI than serum creatinine. In particular, urinary Wnt4 could be a potential noninvasive biomarker for the early detection of tubular injury.

In our study, as expected, we found that the upregulation of kidney Wnt4 occurred at a very early stage (3 hours) after ischemic AKI, as assessed by immunofluorescence and western blot of kidney tissue, at a time when serum creatinine had not significantly increased. These data clearly indicate that the expression of kidney Wnt4 increased during the early phase of AKI, earlier than the serum creatinine. And the tendency of Wnt4 level to change in a time-dependent manner was correlated with the score of renal tubular injury, which was similar to KIM-1. Kidney Wnt4 expression was positively correlated with KIM-1, which was a specific biomarker for kidney tubular injury^21^. These findings confirm that kidney Wnt4 could better reflect tubular injury than the present gold standard indicator serum creatinine.

Next, we investigated the role of Wnt4 in reflecting either the injury phase or regenerative phase in renal tubules after IRI using TUNEL and the pan cell cycle marker Ki67. The number of TUNEL positive apoptotic cells rapidly peaked at 24 hours after IRI and then gradually decreased over time. However, the Ki67 positive cells gradually increased and peaked at 72 hours after IRI, consistent with previous studies^22^,^23^. Furthermore, we found that almost all TUNEL positive cells and majority of Ki67 positive cells costained with Wnt4. In addition, other studies and our previous data demonstrated that the Wnt pathway is critical for kidney repair and regeneration^11^,^13^. Therefore, Wnt4 might be promptly activated after kidney injury and then involved in the repair and regeneration after IRI. Further studies are needed to verify these findings in other AKI models.

In the present study, we also observed enhanced Wnt4 expression in injured tubular epithelial cells (both proximal and distal tubular segments). However, enhanced expression of KIM-1 is simply identified in proximal tubule following kidney injury^17^,^24^. Wnt4 could also be used to detect subtle damage in distal tubule segments, though further studies are required. Therefore, Wnt4 may serve as a better indicator for early detection after kidney injury compared with serum creatinine.

Because the evaluation of urinary indicators is a noninvasive and sensitive method for the estimation of kidney injury and for monitoring the therapeutic effects of an intervention, which is a requirement for an ideal early biomarker of AKI, we further investigated whether urinary Wnt4 could be detected at early stage after IRI. First, we detected the excretion of urinary Wnt4 in IRI mice and sham-operated mice. As expected, urinary Wnt4 level (both western blot and ELISA) increased earlier, at 3 hours after IRI, than serum creatinine. The urinary Wnt4 excretion was strongly related to the severity of kidney tubular injury and kidney Wnt4 expression. These results confirmed that urinary Wnt4 might be an early biomarker of AKI. Its excretion into the urine gives Wnt4 the potential to be an early biomarker after kidney IRI, although the definite mechanisms require further study.

To further investigate this observation, we extended our experiment to clinical patients. According to KIDGO Guidelines, serum creatinine combined with urine output will remain the cornerstone for diagnosing AKI. However, serum creatinine is a late and poor indicator for AKI^5^. A rise in serum creatinine is often a sign of severe kidney damage, even if the rise in creatinine is minimal, and is therefore more indicative of dysfunction rather than damage. Many studies with several thousands of patients have shown the additional value of new biomarkers not only because they enable earlier diagnoses^25^ but also because they allow kidney injury to be diagnosed even in the absence of subsequent signs of dysfunction^26^,^27^. In addition, histopathological criteria are difficult to apply to the diagnosis of AKI in patients in whom kidney biopsy is usually considered to carry an inappropriate risk-to-benefit ratio. AKI is a common complication of MCD patients^28^, and of all the biopsy-proven patients, we found that some MCD patients displayed tubular injury without a corresponding increase in serum creatinine. Van Timmeren *et al*.^29^ reported that KIM-1 expression significantly increased in the apical side of dilated tubules in fibrotic areas in all kidney diseases except MCD and that KIM-1-positive tubular cells all exhibited a dedifferentiated phenotype^30^. We therefore selected 40 MCD patients with or without tubular injury, as diagnosed by renal tissue biopsy, all of whom had normal serum creatinine. In the biopsy specimens from the MCD patients with tubular injury, although serum creatinine was not increased, we observed marked injury in the renal tubules, such as tubular swelling and deformation, expansion, tubular epithelial cell degeneration and necrosis, and the accumulation of necrotic material within the lumen. Immunohistochemistry demonstrated that Wnt4 and KIM-1 were upregulated in the renal tubular epithelial cells of the MCD with tubular injury group compared to MCD without tubular injury patients, although the eGFR and serum creatinine were not significantly different between the groups. In addition, the increased Wnt4 expression was strongly correlated with histopathological alterations and KIM-1 expression in MCD patients. It was encouraging that similar results were obtained in the urine, as urinary Wnt4 was detected in MCD patients with tubular injury. Furthermore, urinary Wnt4 increased substantially and correlated with the degrees of renal expression and renal tubular injury despite no difference in serum creatinine between the two groups, which was similar to urinary KIM-1 excretion. KIM-1 expression identifies proximal tubular injury sensitively and specifically^29^. This work focused on the investigation of a novel AKI biomarker for diagnosing AKI before a marked increase in serum creatinine or a decrease in urine output, which are the currently accepted gold standards^31^. We demonstrated that Wnt4 excretion in the urine was upregulated in the early phase of ischemic AKI in mice and in clinical MCD patients with tubular injury unaccompanied by an increase in serum creatinine. In addition, urinary Wnt4 levels positively correlated with urinary KIM-1 expression in both IRI mice and MCD patients. These results demonstrate that normalized urinary Wnt4 levels may be an attractive novel biomarker for the early detection of AKI. One of the difficulties in using AKI biomarkers has been to identify which patients would benefit most from their use. Therefore, Wnt4 requires further validation as a novel biomarker of AKI and further clinical and experimental studies with long-term follow-up of these MCD patients are needed.

Further studies will be required to fully assess the patterns of elevation in kidney and urinary Wnt4 using other kidney-injury models. In addition, we need to investigate whether urinary Wnt4 can serve as a noninvasive efficacy biomarker for evaluating the extent of kidney injury and to predict outcomes. Another limitation of this study is the small sample size of the clinical study. Further studies using large cohorts with AKI induced by different mechanisms are therefore needed.

In conclusion, this is the first demonstration that both increased kidney and urinary Wnt4 expression can be detected earlier than serum creatinine after IRI. Especially, urinary Wnt4 could be a potential noninvasive biomarker for the early detection of tubular injury.

## Methods

### Animals and animal models

Male C57BL/6 mice purchased from the 2^nd^ Affiliated Hospital Laboratories of Harbin Medical University were used at an age of 8 to 10 weeks and a weight between 20 and 24g. The mice were subjected to kidney IRI by methods described previously (bilateral model of 20 minutes of ischemia)^2^. Age- and weight-matched male C57BL/6 mice that received sham operations with the same manipulation of the pedicle but without clamping of the renal arteries served as normal controls. All experimental procedures and animal care protocols were approved by the animal committee of Harbin Medical University. Animal experiments were performed in strict accordance with the Health Guidelines of the National Institutes for the Care and Use of Laboratory Animals. Mice (n = 8 in each group) were sacrificed under anesthesia (1% pentobarbital, 60 mg/kg body weight, intraperitoneal injection), and all efforts were made to minimize pain and discomfort.

### Clinical patients and parameter measurements

All of the patients who were recruited for this study were admitted to the Nephrology Department in the 2^nd^ Affiliated Hospital of Harbin Medical University. Human kidney tissues were collected during renal biopsies from patients with newly diagnosed nephrotic syndrome (defined as 24-hour urinary protein > 3.5 g/d, serum albumin < 30 g/L according to the KDIGO criteria). MCD refers to the occurrence of nephrotic syndrome with no glomerular lesions by light microscopy (or only minimal mesangial prominence), no staining on immunofluorescence microscopy (or low-intensity staining for C3 and IgM), and foot process effacement but no electron-dense deposits on electron microscopy. The duration time between the first visits of these patients and renal biopsy was about 2 days. Urinary samples were collected from the patients in the same day of renal biopsy and stored at −20 °C within 2 hours of collection until creatinine, Wnt4 and KIM-1 levels were analyzed. The Internal Review Board of Harbin Medical Hospital approved the study protocol, and all patients provided informed consent according to the latest version of the Helsinki Declaration on human research ethics. All methods were carried out in accordance with the approved guidelines.

### Sample collection and serum parameter measurements

IRI mice were housed in an air-conditioned room and were given free access to food and water (22 ± 2 °C; 12:12 hour light dark cycle) (n = 8 mice in each group). At 0, 3, 6, 12, 24, 72, 120 and 168 hours after bilateral IRI, urine and serum were collected and stored at −20 °C until further analysis. Urine was collected before euthanasia from each mouse housed in metabolic cages with free access to water but without food. Blood samples were collected from the retrobulbar plexus of anesthetized mice. The urine and blood samples were centrifuged for 10 minutes at 3500 rpm. The supernatants were collected and stored at −20 °C until analysis. Plasma creatinine, urine creatinine and plasma BUN were measured using the enzymatic method by standard laboratory techniques with an automatic biochemistry analyzer (Cobasc 311, Roche, Germany) as previously reported^32^,^33^,^34^. Renal tissues were prepared for immunofluorescence microscopy, morphological studies, and molecular biology experiments.

### Histological studies

Mice were perfused with sterile ice-cold phosphate-buffered saline (PBS). The tissue used for light microscopy was fixed in 10% neutral-buffered formalin for 12 hours, dehydrated in graded ethanol, embedded in paraffin for sectioning (2–3 μm and stained with HE, PAS, and Masson’s trichrome. Images were captured using a Nikon DS Ri1 (Tokyo, Japan).

### Immunofluorescence staining

Mice were perfused with sterile ice-cold PBS. Kidney tissues were fixed in paraformaldehyde/lysine/periodate (PLP) solution for 2 hours, followed by 18% sucrose overnight. The tissues were then embedded in Tissue-Tek O.C.T. compound (stored at −80 °C). Frozen sections were obtained using a cryostat (Thermo Scientific, Cheshire, UK) at 4 μm. Immunofluorescence labeling was performed using a previously described protocol^2^,^11^. Primary antibodies against the following proteins were used for immunostaining: rabbit anti-mouse Wnt-4 (1:200, Santa Cruz Biotech, Delaware Avenue, CA, USA), goat anti-mouse KIM-1 (1:400, R&D Systems, Minneapolis, MN, USA), goat anti-mouse Ki67 (1:200, Santa Cruz Biotech, Delaware Avenue, CA, USA), goat anti-mouse AQP1 (1:200, Santa Cruz Biotech, Delaware Avenue, CA, USA), goat anti-mouse NCC (1:200, Santa Cruz Biotech, Delaware Avenue, CA, USA). The secondary antibodies were Alexa Fluor 594/488-conjugated donkey anti-rabbit, Alexa Fluor 488-conjugated donkey anti-goat antibodies (1:200; Jackson ImmunoResearch Laboratories, West Grove, PA). Nuclei were stained using 4, 6-diamidino-2-phenylindole (DAPI). Images were captured using a Nikon DS Ri1 (Tokyo, Japan).

### Immunohistochemical staining

Immunohistochemical staining and Ki-67 staining of paraffin sections was performed by dewaxing, antigen retrieval (citric acid buffer incubation and microwave heating), and antibody incubation as previously described^35^. Briefly, paraffin sections were obtained using a microtome (Thermo Scientific, Walldorf, Germany) at 2–3 μm. The sections were deparaffinized, and endogenous peroxidase activity was ablated by incubation in 3% hydrogen peroxide in methanol for 5 minutes. The sections were then heated in a microwave oven in 0.1 mol/L citrate buffer (pH 6.0) for 20 min. Rabbit anti-mouse Wnt-4 antibody (1:200, Santa Cruz Biotech, Delaware Avenue, CA, USA) was then added to the sections and incubated overnight at 4 °C. The secondary antibody was horseradish peroxidase-conjugated goat anti-rabbit IgG (BOSTER, Wuhan, China), and coloration was performed with a DAB Kit (BOSTER, Wuhan, China).

### TUNEL staining

Apoptotic cells were detected using a TUNEL apoptosis assay kit (Roche, Indianapolis, IN, USA) according to the manufacturer’s protocol. Images were captured using a Nikon microscope (Tokyo, Japan) and processed using NIS-Elements software (Tokyo, Japan).

### Images capture and quantification

Images were sequentially captured by digital imaging (200x/400x magnification) at the cortical and outer medullary regions (10–15 images). All exposure settings were the same for each group of kidneys. The data obtained from each tissue are represented as the mean of all fields. The degree of tubular injury was assessed as previously reported^2^. Each image of mice and human was divided into 252 squares using a grid. To calculate the presence of tubule injury in the square, each square with tubular injury (tubule flattening, brush-border loss, necrosis, apoptosis or cast formation) was assigned a positive score. The final score is presented as a percentage positive score, which was averaged for all images from the individual kidney. Images were captured using a Nikon microscope (Tokyo, Japan). The positive immunofluorescent or immunohistochemical area (Wnt4 and KIM-1) was measured by manually outlining the perimeter of ten images in each section, and the mean area of the outlined regions (positive staining) was quantified and processed using image analysis software (ImageJ, version 1.47, National Institutes of Health, USA). The number of Ki-67 positive cells and TUNEL positive cells were counted from 10 different fields for each sample and averaged.

### Western blot analysis

Kidney tissues were lysed on ice with a radioimmunoprecipitation assay (RIPA) buffer containing 1% NP-40, 0.1% SDS, 100 μg/ml PMSF, 1% protease inhibitor cocktail, and 1% phosphatase I and II inhibitor cocktail (Sigma). The same amount of kidney total protein lysates (200 μg) or centrifuged urine (100 μg) were denatured at 80 °C for 10 minutes in sample buffer, separated in a 12% polyacrylamide sodium dodecyl sulfate gel, and transferred onto a PVDF membrane. The PVDF membranes were blocked at room temperature for 2 hours with 5% powdered milk in Tris-HCl buffer containing 0.1% Tween 20 (TBST). The western blot analysis was carried out as previously described^36^. To detect Wnt4 and KIM-1, the blots were incubated with rabbit anti-mouse/human Wnt4 (1:200, Santa Cruz Biotech, Delaware Avenue, CA, USA), goat anti-mouse/human KIM-1 (1:400, R&D Systems, Minneapolis, MN, USA) and then with HRP-conjugated goat anti-rabbit IgG, donkey anti-goat or goat anti-mouse IgG antibody (1:5000, Jackson ImmunoResearch Laboratories, West Grove, PA, USA). Renal western blot results were normalized to β-actin.

### Enzyme-linked immunosorbent assay (ELISA)

Urinary Wnt4 and KIM-1 from IRI mice and MCD patients were measured by enzyme-linked immunosorbent assay (ELISA) using a commercially available test kit (CUSABIO, Wuhan, China) according to the manufacturer’s protocol and were normalized to urinary creatinine (uCr).

### Statistical analyses

Statistical analysis was performed using the GraphPad Prism Software, version 6.0. The results are presented as the mean ± standard deviation (SD). One-way ANOVA with a Steel-Dwass test was used for determining differences between groups. Kaplan-Meier analysis was used to analyze survival rate. Comparisons between two groups were made using an unpaired t-test. Correlations were determined by two-tailed Pearson correlation analysis. The correlation coefficient was applied as the index of measuring correlation. P values less than 0.05 were considered significant for all statistical tests.

## Additional Information

**How to cite this article**: Zhao, S.-L. *et al*. Wnt4 is a novel biomarker for the early detection of kidney tubular injury after ischemia/reperfusion injury. *Sci. Rep.* **6**, 32610; doi: 10.1038/srep32610 (2016).

## Supplementary Material

Supplementary Information

## Figures and Tables

**Table 1:** Baseline characteristics of the enrolled patients.

	**All patients (MCD)**	**MCD only**	**MCD with tubular injury**	**P value**
No. of patients included	40	21	19	
Age (years)	45.4 ± 16.7	41.5 ± 16.2	53.2 ± 15.8	0.952
Male/female ratio	23/17	13/8	10/9	
Weight (kg)	70.1 ± 11.8	69.5 ± 11.3	70.1 ± 11.8	0.437
24-h urine protein (g/d)	11.5 ± 7.9	12.5 ± 9.8	10.4 ± 3.9	0.688
Serum albumin (g/L)	18.7 ± 7.1	20.0 ± 7.5	16.1 ± 5.8	0.533
Serum creatinine (μmol/L)	72.1 ± 21.0	67.5 ± 17.7	77.1 ± 23.7	0.354
BUN (mmol/L)	5.5 ± 2.5	5.9 ± 2.4	5.0 ± 2.6	0.451
Cholesterol (mmol/L)	9.8 ± 3.0	10.0. ± 3.3	9.6 ± 2.6	0.914
Blood pressure (mmHg)	132.1 ± 20.0/79.1 ± 11.6	130.1 ± 22.2/79.1 ± 11.7	136.0 ± 15.1/79.2 ± 12.2	0.732/0.983
eGFR (ml/min/1.73 m^2^)	87.9 ± 24.9	92.1 ± 27.6	83.4 ± 21.8	0.549

The number of patients in each group is noted on the chart. The chart indicates the mean ± standard deviation of measurements of selected physical and biochemical data from the MCD patients with and without tubular injury.
