# Supplementary material for: Wnt4 is a novel biomarker for the early detection of kidney tubular injury after ischemia/reperfusion injury
Source: Sci Rep. 2016 Sep 7;6:32610. doi: 10.1038/srep32610 (PMC5013493; doi:10.1038/srep32610)
Supplement: Supplementary Information [file srep32610-s1.pdf]

Wnt4 is a novel biomarker for the early detection of kidney tubular injury after ischemia/reperfusion injury

SHI-LEI ZHAO[1,2] M.D., SHI-YAO WEI[1] M.D., YU-XIAO WANG[1] M.D., TIAN-TIAN DIAO[1] M.D., JIAN-SI LI[1] M.D., YI-XIN HE[1] M.D., JING YU[1] M.D., XI-YUE JIANG[1] M.D., YANG CAO[1] M.D., XIN-YUE MAO[1] M.D., QIU-JU WEI[1] M.D., YU WANG[1] M.D., BING LI[1]* M.D., PhD.

[1]*Department of Nephrology, 2nd Affiliated Hospital;* [2]*Department of Nephrology, 1st Affiliated Hospital, Harbin Medical University, Harbin, People's Republic of China.*

Running title: Wnt4 is a novel biomarker of AKI

Correspondence to: Dr. Bing Li, Department of Nephrology, 2nd Affiliated Hospital of Harbin Medical University, 246 Xuefu Road, Nangang District, Harbin 150086, P.R.C. Telephone: 0086-451-86297145. E-mail: icecreamlee@hotmail.com

Key words: Wnt4, kidney tubular injury, ischemia/reperfusion injury, biomarker.

Supplementary Figures and Figure Legends

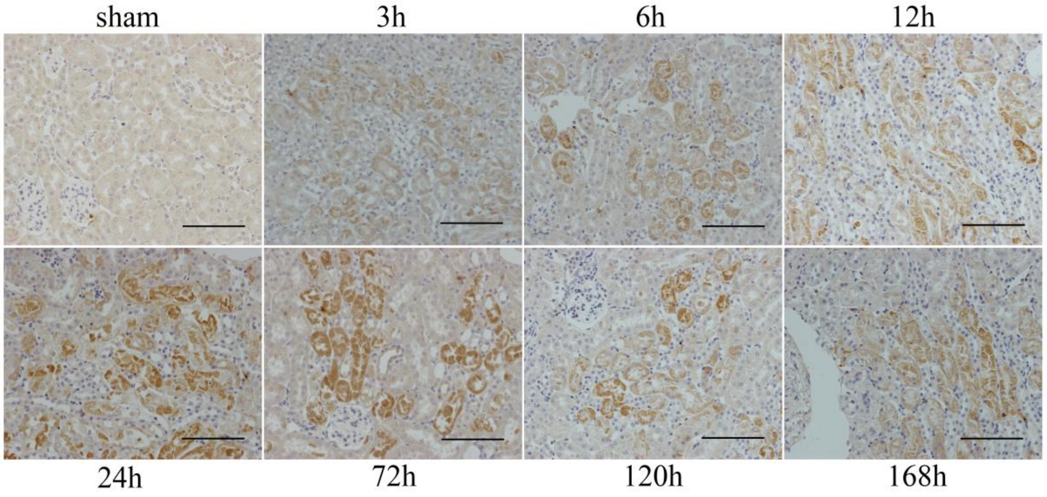

Supplementary Figure S1. Representative immunohistochemical images of kidney Wnt4 in IRI mice at different time points and in sham-operated mice (magnification, 200x). Wnt4 expression slightly increased at 3 hours and peaked at 24 hours in the injured tubules after IRI. Scale bar, 100 μm.

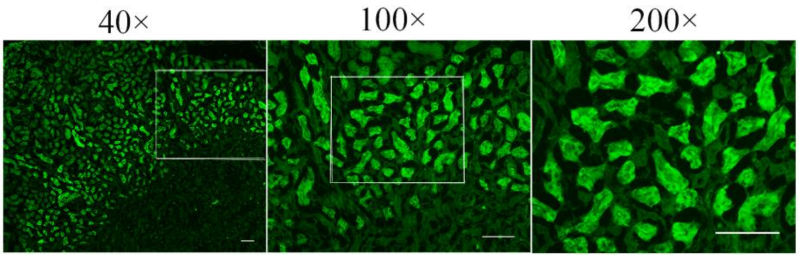

Supplementary Figure S2. The major location of Wnt4 expression was in the outer medulla (Magnification, 40x, 100x, 200x). Scale bar, 100 μm.

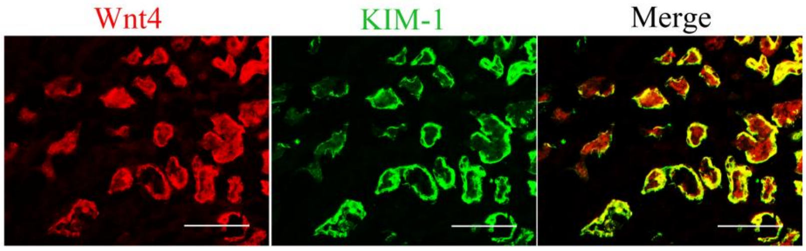

Supplementary Figure S3. Co-staining for Wnt4 and KIM-1 in injured tubules 12 hours after IRI (magnification, 200x). Wnt4 and KIM-1 mostly co-localized in the injured proximal tubules. Scale bar, 100 μm.

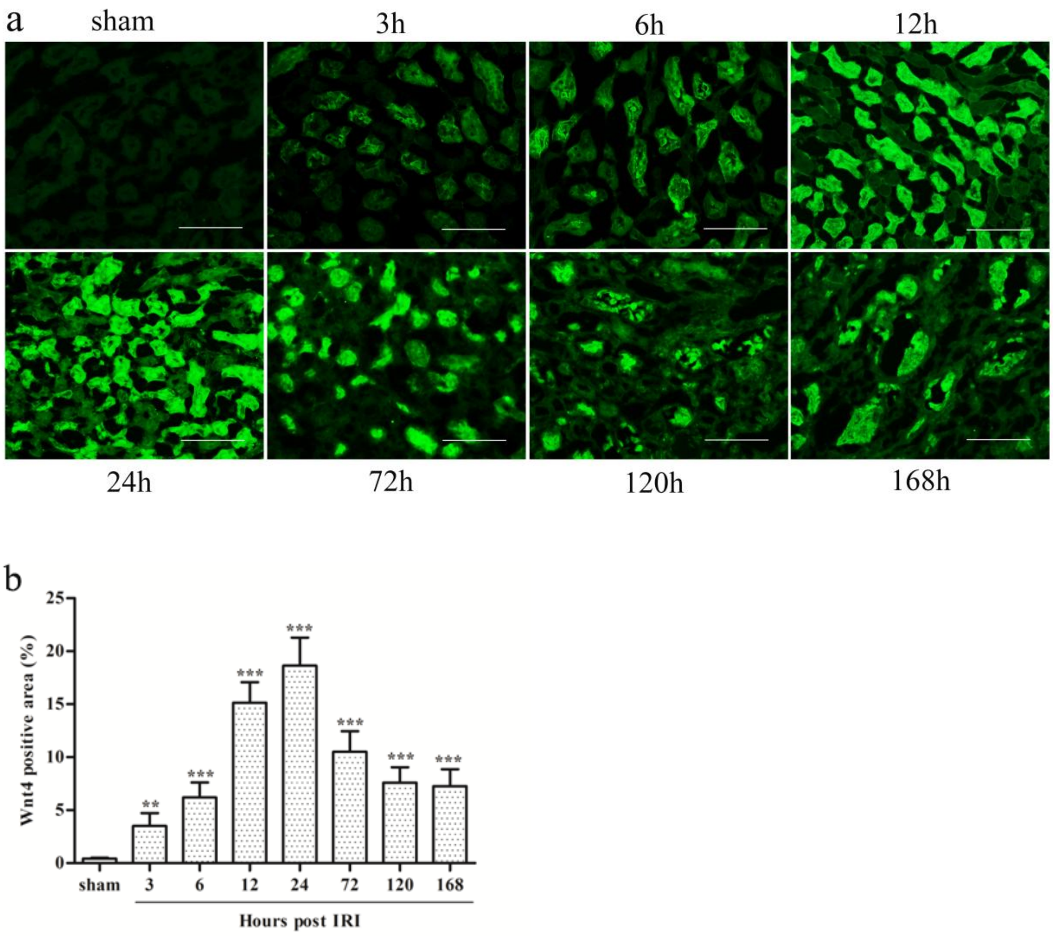

Supplementary Figure S4. a. Representative immunofluorescence images of kidney Wnt4 in 25-minute IRI mice at different time points and in sham-operated mice

(magnification, 200x). As shown in the figure, enhanced Wnt4 expression occurred in injured tubules as early as 3 hours after IRI and reached a peak at 12 and 24 hours that persisted for at least 7 days after IRI. Scale bar, 100 μm. **b**. Quantification of kidney Wnt4 expression in 25-minute IRI mice. **P<0.01, ***P<0.001 versus the sham group (n=8).

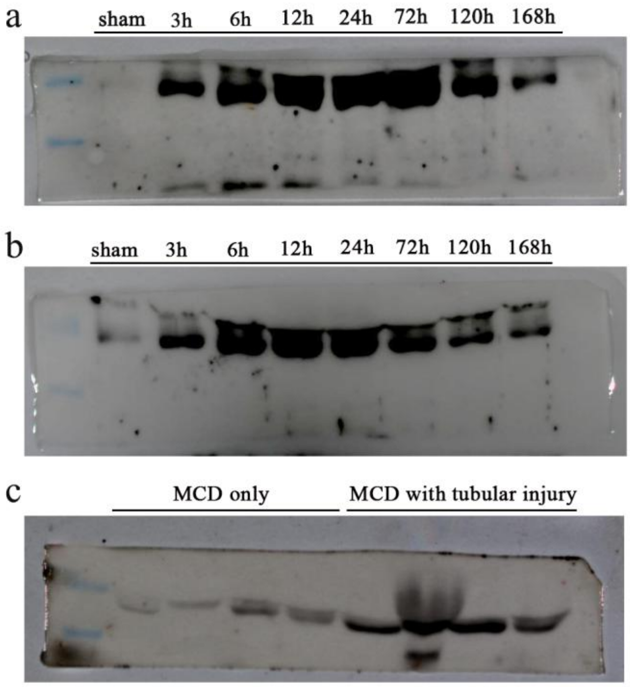

Supplementary Figure S5. Full-sized blots are shown. **a**. Kidney Wnt4 expression in mice. **b**. Urinary Wnt4 expression in mice. **c.** Urinary Wnt4 expression in MCD patients.
